# Highly sensitive ELISA‐based assay for quantification of allergen‐specific IgE antibody levels

**DOI:** 10.1111/all.14325

**Published:** 2020-05-27

**Authors:** Antonina Karsonova, Ksenja Riabova, Sergio Villazala‐Merino, Raffaela Campana, Verena Niederberger, Julia Eckl‐Dorna, Renate Fröschl, Thomas Perkmann, Yury V. Zhernov, Olga G. Elisyutina, Elena S. Fedenko, Musa R. Khaitov, Daria Fomina, Evgeniy Beltiukov, Marianne van Hage, Hans Grönlund, Rudolf Valenta, Alexander Karaulov, Mirela Curin

**Affiliations:** ^1^ Laboratory of Immunopathology Department of Clinical Immunology and Allergy Sechenov First Moscow State Medical University Moscow Russian Federation; ^2^ Department of Otorhinolaryngology Medical University of Vienna Vienna Austria; ^3^ Division of Immunopathology Department of Pathophysiology and Allergy Research Center for Pathophysiology, Infectiology and Immunology Medical University of Vienna Vienna Austria; ^4^ Department of Laboratory Medicine Medical University of Vienna Vienna Austria; ^5^ National Research Center ‐ Institute of Immunology FMBA of Russia Moscow Russian Federation; ^6^ City Hospital #52 Moscow Russian Federation; ^7^ Ural State Medical University Ekaterinburg Russian Federation; ^8^ Division of Immunology and Allergy Department of Medicine Solna Karolinska Institutet and Karolinska University Hospital Stockholm Sweden; ^9^ Department of Clinical Neuroscience Therapeutic Immune Design Unit Karolinska Institutet Stockholm Sweden; ^10^ Karl Landsteiner University for Healthcare Sciences Krems Austria

To the Editor,

Allergy is the most common immunologically mediated hypersensitivity disease worldwide affecting upper and lower respiratory tract, the skin as well as the gastrointestinal tract and may cause life‐threatening anaphylaxis.^1^ Immunoglobulin E (IgE) occurs in extremely low amounts in blood and tissues but is the key immunoglobulin in allergy. The quantification of allergen‐specific IgE levels (sIgE) in serum and body fluids is important because sIgE concentrations are useful to define clinical phenotypes of allergy and to predict the intensity of allergic reactions.^2^, ^3^, ^4^, ^5^ However, quantification of sIgE requires special instruments (eg, ImmunoCAP) that are available only in industrialized countries, large volumes of serum and is quite expensive.^6^ Therefore, the goal of our study was to establish a quantitative method for measuring allergen‐specific IgE based on a simple ELISA technology which can be implemented and used in all parts of the world. The user will thus need only equipment for ELISA, reagents, detection antibodies and test allergens plus reagents for the IgE standard curve.

In order to establish a standard for ELISA‐based quantification of allergen‐specific human IgE, a human monoclonal chimeric IgE antibody (IgEmoAb)^7^ consisting of the variable region of a mouse IgG_1_ antibody specific for the major birch pollen allergen Bet v 1, which had been fused to the human epsilon heavy chain, was expressed and purified from hybridoma cells by affinity chromatography to >95% purity (Figure S1A). Under non‐reducing conditions, purified IgEmoAb migrates as a distinct single band of approximately 250 kDa molecular weight in SDS‐PAGE, while under reducing conditions, two distinct bands which correspond to the heavy and light chain became visible at approximately 100 and 20 kDa, respectively (Figure S1A). IgEmoAb reacted specifically with anti‐human IgE antibodies under non‐reducing conditions, and under reducing conditions, the heavy chain showed selective anti‐IgE reactivity by immunoblotting (Figure S1B). Structural analysis of IgEmoAb by circular dichroism showed that it assumed a predominant beta‐sheet fold with a minimum signal around 217 nm and a maximum near 198 nm (Figure S1C).

Using IgEmoAb and Bet v 1, we next established by ELISA a standard curve allowing to measure and quantify sIgE. A series of dilutions of IgEmoAb ranging from 2.42 to 2420 ng/mL was exposed to ELISA plate‐bound IgE yielding standard curves with a linear range of approximately 2.42‐242 ng/mL IgE depending on the time of assay development (Figure 1A). Figure S2 shows the OD values obtained for the dilution series of IgEmoAb determined by ELISA at different time points of assay development, that is, (after 5, 10, 15, and 20 minutes) yielding optimal results from 5 to 10 minutes of development with the given detection antibodies and color reagents. The different dilutions of IgEmoAb were also measured using Streptavidin ImmunoCAPs prepared with the same recombinant Bet v 1 preparation used in the ELISA experiments. The concentrations of IgEmoAb, that is, 2.42, 24.2, 242, and 2420 ng/mL corresponded to the following ImmunoCAP results: 0.34, 3.28, 33.1, and >100 kU_A_/L showing that the dilutions of the IgEmoAb standard were correct down to few nanograms per mL without loss of activity. However, we noted that the ImmunoCAP determination of the concentrations of IgEmoAb was different (ie, yielding approx. 30%) from the results obtained by microBCA protein determination which may be due to differences between the ImmunoCAP IgE standard and IgEmoAB (data not shown). Accordingly, a “real conversion factor” of 7 ng/mL = 1 kUA/L for calculating IgE concentrations had to be introduced.

**Figure 1:**
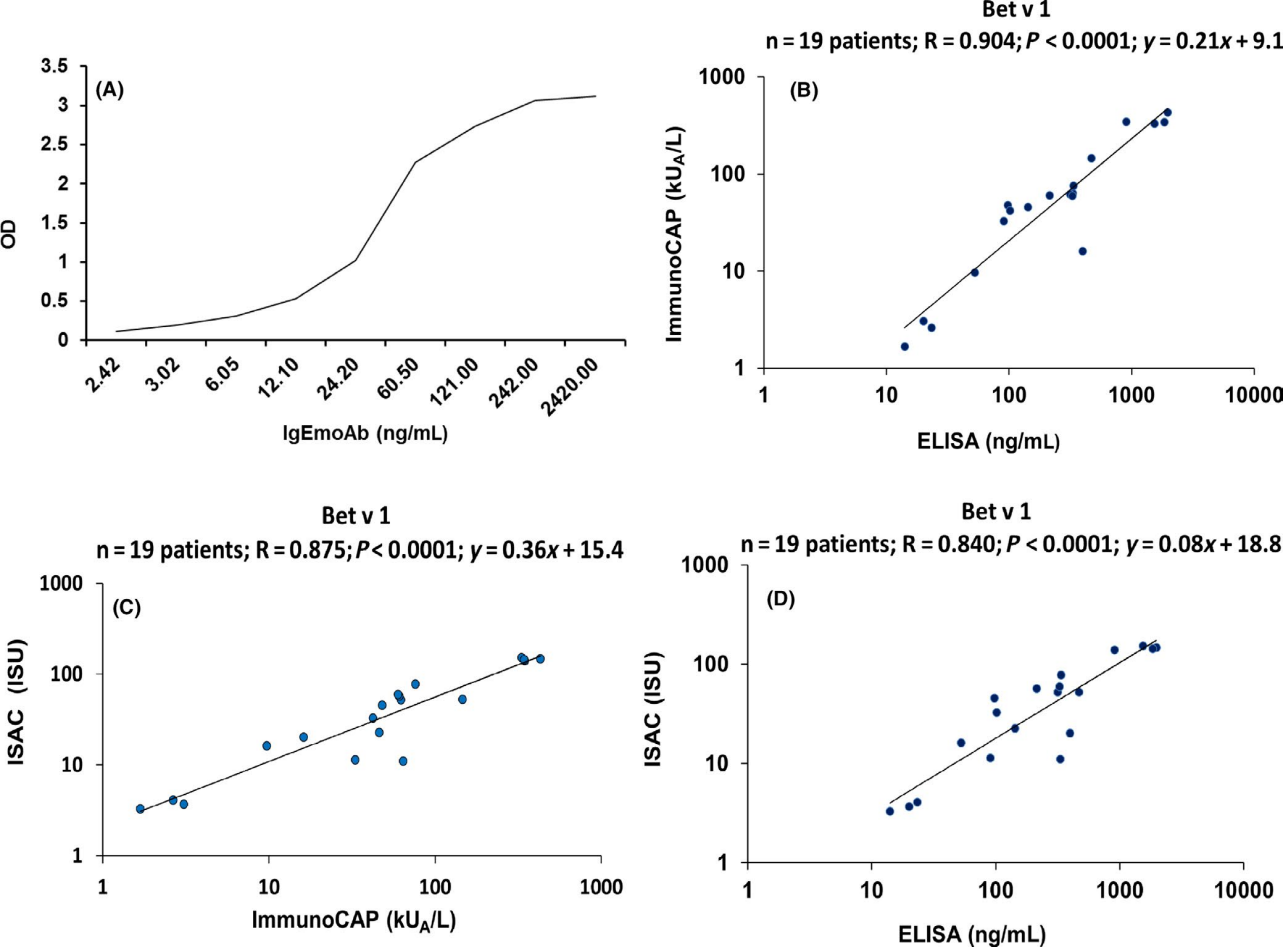
Association of different concentrations of IgEmoAb (x‐axis: ng/mL) with IgE reactivities to Bet v 1 determined as optical density (OD) values (y‐axis) by ELISA (A). Correlation of Bet v 1‐specific IgE levels determined in sera from 19 birch pollen allergic patients by different methods. B, ImmunoCAP (kU_A_/L) (y‐axis) versus ng/ml determined by ELISA (x‐axis). C, ISU (ISAC standardized unit) values determined by ImmunoCAP ISAC (y‐axis) vs ImmunoCAP (kU_A_/L) (x‐axis) and D, ISU values determined by ImmunoCAP ISAC (y‐axis) vs ng/mL determined by ELISA (x‐axis). Correlation coefficients (*r*), regression equations, and statistical significances (*P* values) are shown in the top upper corner of graph

Based on a standard curve established with IgEmoAb, Bet v 1‐specific IgE concentrations were quantified in 19 sera from clinically well‐characterized birch pollen allergic patients by ELISA (ng/ml) (Tables S1 and S2). Table S2 displays the OD levels, the corresponding ng/mL concentrations of Bet v 1‐specific IgE, and the results from the ImmunoCAP and ImmunoCAP ISAC measurements in detail. We noted a strong significant correlation between the Bet v 1‐specific IgE concentrations determined in ng/ml with the ELISA and in kU_A_/l ImmunoCAP results (*r* = .904, *P* < .0001, 95% CI 0.756‐0.964; Figure 1B). In fact, Bet v 1‐specific IgE levels measured by ELISA correlated even better with ImmunoCAP results than IgE levels measured with ImmunoCAP ISAC chip technology (*r* = 0.875, *P* < .0001, 95% CI 0.692‐0.953; Figure 1C). Nevertheless, results of the quantitative IgE ELISA correlated also significantly with sIgE levels measured with ImmunoCAP ISAC chip technology (*r* = .840, *P* < .0001, 95% CI 0.616‐0.939) (Figure 1D). In summary, strong correlations of Bet v 1‐specific IgE levels measured by ELISA with those determined by ImmunoCAP and ImmunoCAP ISAC technology were obtained.

Next, we investigated whether the IgEmoAb‐based standard curve is also suitable for the quantification of patients' IgE to an allergen molecule unrelated to Bet v 1. For this purpose, we selected the major cat allergen Fel d 1 which besides Bet v 1 is one of the most frequently recognized allergens in the Russian population.^5^, ^8^ Quantitative IgE ELISA was used to determine Fel d 1‐specific IgE levels in 19 cat allergic patients using different serum dilutions. Detailed results of quantitative IgE ELISA and of ImmunoCAP are shown in Tables S3 and S4. A strong significant correlation was obtained between Fel d 1‐specific IgE concentrations quantified with the IgE ELISA (ng/mL) and with the ImmunoCAP results (kU_A_/L) (*r* = .984, *P* < .0001, 95% CI 0.956‐0.994; Figure 2A) demonstrating that the quantitative ELISA can be used for quantification of specific IgE levels against different allergens, for example, Fel d 1. Interestingly, we noted for three patients with clinically confirmed symptoms of cat allergy ImmunoCAP measurements were below the threshold of 0.1 kU_A_/L but we were able to detect and quantify Fel d 1‐specific IgE with the IgE ELISA (Table S3, patients C4, C5, and C12). Of note, we found that the results obtained for different dilutions of the sera delivered highly comparable results. Results of IgE level assessment obtained from 1:2 sera dilutions and 1:4 sera dilutions from the cat allergic patients (shown in Table S4) were compared, and a strong and significant correlation was noted (*r* = .988 *P* < .0001, 95% CI 0.967‐0.996; Figure 2B).

**Figure 2:**
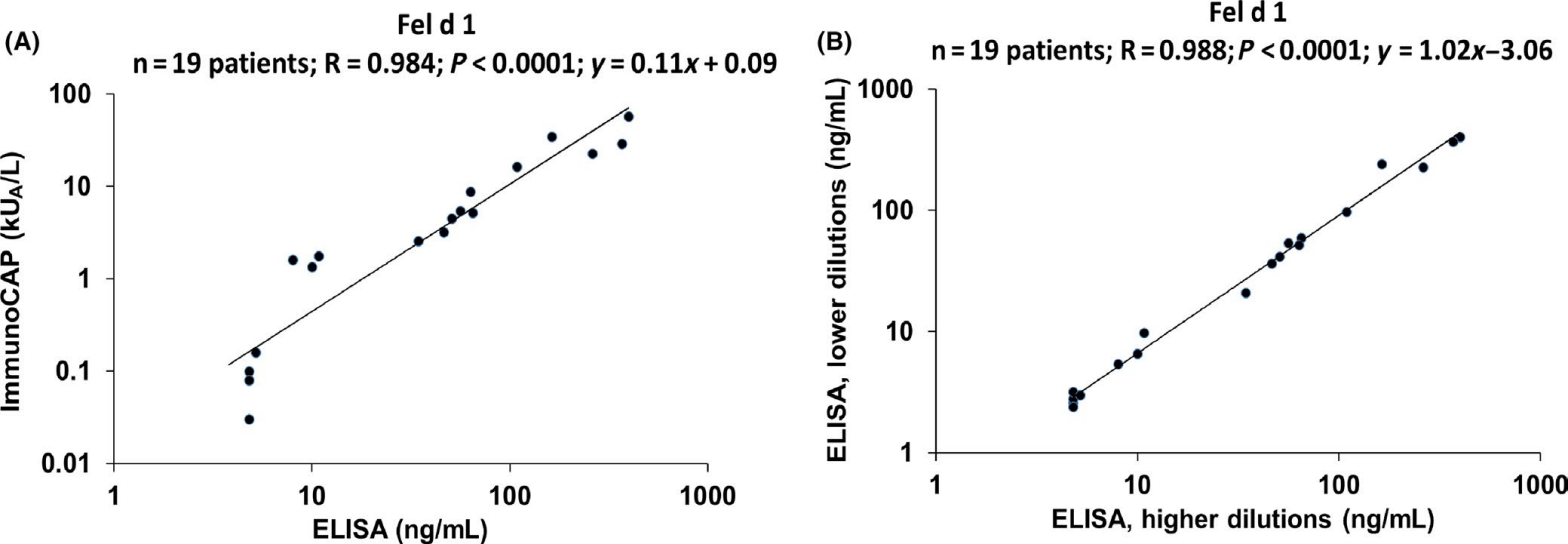
Correlation of Fel d 1‐specific IgE levels determined in sera from 19 cat allergic patients by ImmunoCAP (kU_A_/L) (y‐axis) versus ng/ml determined by ELISA (x‐axis) (A). Correlation of Fel d 1‐specific IgE levels determined in lower (y‐axis) and higher (x‐axis) sera dilutions from 19 cat allergic patients from Table S3 (B). Correlation coefficients (*r*), regression equations, and statistical significances (*P* values) are shown in the top upper corner of graph

The quantitative IgE ELISA assay should be useful for laboratories in less developed countries which cannot afford expensive IgE test systems for routine allergy diagnosis taking advantage of molecular allergy diagnosis.^9^, ^10^ Before extensive use, broader clinical studies correlating IgE concentrations to clinical signs may be warranted. In fact, the price for a quantitative IgE determination for one allergen should cost less than 10% than by ImmunoCAP; however, the time required for testing will be longer. Our assay will therefore be useful in places where work power cost is not a major issue and the results are desired in a higher‐throughput format as well as for cases (eg, research) where regulations are of minor importance. Furthermore, the assay should allow a flexible testing of different allergen molecules which can be kept on stock and used depending on the clinical questions and diagnostic needs required. To achieve these goals, the authors will make efforts to make available the key reagents for the standard curve (ie, IgEmoAb and rBet v 1) to the community. In summary, we provide a simple, inexpensive, sensitive and accurate method for measuring allergen‐specific IgE concentrations in serum and body fluids to facilitate the diagnosis of IgE‐associated allergy in the world.

## CONFLICT OF INTEREST

RV has received research grants from HVD Life Sciences, Vienna, Austria and Viravaxx, Vienna, Austria, and serves as consultant for Viravaxx. MvH has received lecture fees from Thermo Fisher Scientific and consultancy fees from Biomay AG, Vienna, Austria, and Hycor Biomedical LLC, CA, US, outside of the submitted work. Other authors have no conflict of interest to declare.

## Supporting information

Supplementary Material
