# Supplementary material for: Highly sensitive ELISA‐based assay for quantification of allergen‐specific IgE antibody levels
Source: Allergy. 2020 May 27;75(10):2668–70. doi: 10.1111/all.14325 (PMC7687237; doi:10.1111/all.14325)
Supplement: Supplementary file 1 — Supplementary Material [file ALL-75-2668-s001.pdf]

Highly sensitive ELISA-based assay for quantification of allergen-specific IgE antibody levels

Antonina Karsonova[1], Ksenja Riabova[1], Sergio Villazala-Merino[2], Raffaela Campana[3], Verena Niederberger[2], Julia Eckl-Dorna[2], Renate Fröschl[4], Thomas Perkmann[4], Yury V. Zhernov[5], Olga G. Elisyutina[5], Elena S. Fedenko[5], Musa R. Khaitov[5], Daria Fomina[1,6], Evgeniy Beltiukov[7], Marianne van Hage[8], Hans Grönlund[9], Rudolf Valenta[3,10], Alexander Karaulov[1], Mirela Curin[3]

[1]Laboratory of Immunopathology, Department of Clinical Immunology and Allergy, Sechenov First Moscow State Medical University, Moscow, Russian Federation. [2]Department of Otorhinolaryngology, Medical University of Vienna, Vienna, Austria. [3]Division of Immunopathology, Department of Pathophysiology and Allergy Research, Center for Pathophysiology, Infectiology and Immunology, Medical University of Vienna, Vienna, Austria [4]Department of Laboratory Medicine, Medical University of Vienna, Vienna, Austria. [5]National Research Center - Institute of Immunology FMBA of Russia, Moscow, Russian Federation. [6]City Hospital #52, Moscow, Russian Federation. [7]Ural State Medical University, Ekaterinburg, Russian Federation. [8]Division of Immunology and Allergy, Department of Medicine Solna, Karolinska Institutet and Karolinska University Hospital, SE-171 77, Stockholm, Sweden. [9]Department of Clinical Neuroscience, Therapeutic Immune Design Unit, Karolinska Institutet, Stockholm, Sweden.[10]Karl Landsteiner University for Healthcare Sciences, Krems, Austria.

Correspondence:

Mirela Curin, Department of Pathophysiology and Allergy Research, Center for Pathophysiology, Infectiology and Immunology, Medical University of Vienna, Währinger Gürtel 18-20, A-1090 Vienna, Austria. Email: mirela.curin@meduniwien.ac.at

Methods

Expression, purification and characterisation of human allergen-specific monoclonal IgE (IgEmoAb), recombinant allergens

In the humanised monoclonal Bet v 1-specific IgE antibody, the variable region of a mouse IgG_1 antibody specific for the major birch pollen allergy (Bet v 1) [S1], was fused to the human epsilon heavy chain and the DNA construct was introduced into hybridoma cells by electroporation[S1, S2]. Hybridoma cells were cultured in CD Hybridomamedium (ThermoScientifc, Uppsala, Sweeden) supplemented with Glutamax ™ (Thermo Scientific) into which IgEmoAb was secreted. A monoclonal anti-human IgE antibody, mAb12 [S2, S3], was used to purify IgEmoAb from cell culture supernatants by single step affinity chromatography. Purified IgEmoAb was dissolved in PBS and kept frozen at -20°C until use. The concentration of purified IgEmoAb was determined by BCA assay (Pierce, Rockford, IL, USA). In order to check the purity of IgEmoAb, 1 and 2 µg purified antibody along with a protein weight ladder (PageRuler prestained Protein Ladder Plus, Fermentas, St. Leon-Rot, Germany) were loaded onto a 15% polyacrylamide gel and separated by SDS-PAGE under non-reducing and reducing conditions, followed by Coomassie Blue staining. Likewise, 10 and 20 ng IgEmoAb were loaded onto a 15% polyacrylamide gel and separated by SDS-PAGE, followed by semi-dried transfer to a nitrocellulose membrane (GE Healthcare, Chicago, IL, USA). Human IgE was detected upon incubation with 1:5000 Anti-human IgE-HRP (Seracare, Milford, MA, USA).

The far UV circular dichroism (CD) spectrum of purified IgEmoAb was determined using a Jasco J-810 spectropolarimeter (Japan Spectroscopic Co., Tokyo, Japan). An aliquot of 400 µL of a solution with a concentration of 200 µg/mL IgEmoAb in 10 mM NaH_2PO_4 (pH=7) was placed in a 1mm path length quartz cuvette and its spectrum was measured from 260 to 190 nm with a

resolution of 0.5 nm at 50 nm/min. The measurement was repeated three times and averaged. Purified recombinant Bet v 1 was obtained from BIOMAY (Vienna, Austria) and rFel d 1 was expressed and purified as described [S4].

Demographic and clinical characterization of allergic patients and non-allergic control subjects

Experiments were conducted with serum samples of clinically well characterized cat (n = 19) and birch (n = 19) allergic patients from the Russian Federation after having obtained informed consent of the patients or patient`s parents in the case of children. Blood was collected at certified medical centers by licensed medical personnel (Institute of Immunology FMBA of Russia, Moscow; City Hospital #52, Moscow, and Ural State Medical University, Ekaterinburg, Russian Federation). The study was approved by the Ethic Committee of NRC Institute of Immunology FMBA of Russia- protocol No. 3, dated 09.03.2017, performed according to declaration of Helsinki and all participants gave their written informed consent. Each patient completed a questionnaire that contained demographic data, such as age and gender and questions about the symptoms that patients experienced in contact with birch and/or cat. Inclusion criteria were reported clinical symptoms of either asthma, rhinitis, conjunctivitis and/or dermatitis upon exposure to cat or birch. Full description of clinical and demographic characteristics of patients with birch and cat allergy, including phenotypes (asthma, rhinitis, conjunctivitis or dermatitis) are shown in Table S1. Representative sera were selected to comprise the range from low to high of allergen-specific IgE levels. Serum from a non-allergic individual was included as control.

Establishment of IgEmoAb-based standard curves for quantification of sIgE by ELISA

Standard curves for the quantification of allergen-specific IgE were established using ELISA plate coated with rBet v 1 and defined dilutions containing different concentrations of IgEmoAb (i.e., 2.42 µg/ml , 242 ng/ml, 121 ng/ml, 60.5 ng/ml, 24.2 ng/ml, 12.1 ng/ml, 6.05 ng/ml, 3.02 ng/ml, 2.42 ng/ml). The dilutions of IgEmoAb were prepared in 2% BSA in 0.5% PBS-Tween buffer to prevent loss of IgEmoAb due to binding to the plastic vials used for preparing the dilutions (Safe-Lock Tubes 1.5 ml Cat. № 0030120086, Eppendorf, Hamburg, Germany). In pilot experiments, different concentrations of rBet v 1 (1µg/ml, 2 µg/ml, 5µg/ml) were used for coating of ELISA plates (96 well F-bottom microplate, Greiner Bio-One GmbH, Germany) and a concentration of 1 µg/ml of Bet v 1 was found to be suitable for obtaining a standard curve. After coating with rBet v 1 in Bicarbonate buffer plates were blocked with 1% BSA in 0.5% PBS-Tween (200 µl/well) for 2.5 h at 37 ^{0}C. Different dilutions of IgEmoAb in TBS containing 0.5% v/v Tween 20 (TBS-T) and 2% BSA were added to the ELISA plates and bound IgE was detected with HRP-coupled goat anti-human IgE antibodies diluted 1 : 2500 (KPL, Gaithersburg, MD). The OD values corresponding to bound antibodies were measured at 405 and 490 nm on ELISA reader CLARIOstar (BMG Labtech, Germany). All determinations were conducted in triplicates, and results were expressed as mean values with a variation coefficient of less than 5%. Based on the ELISA results the standard curve was established allowing to measure and quantify allergen-specific IgE. The equation describing the Standard Curve was determined as $y=-60.302-50.933x-30.103x^3 +60,7969e^x$ using TableCurve 2D Software.

Quantification of sIgE in sera from allergic patients by ELISA

For coating the ELISA plates, 1µg/ml of rBet v 1 or 1µg/ml of rFel d 1 were incubated in Bicarbonate buffer, (100µl/well) overnight at 4°C. After the plates were washed 3 times with

200µl/well of PBS-Tween unspecific bindings were blocked with 1%BSA in PBS-Tween (200µl/well) for 2.5h, on 37°C. Patient`s serum diluted in 2% BSA-PBS-Tween (dilutions as described below) and set of dilutions of IgEmoAb were added (100µl/well) and incubated at 4°C overnight. Plates were then washed 5 times with 200µl/well of PBS-Tween. For detection of human IgE antibodies anti-human IgE-KPL (coupled with horse radish peroxidase) in dilution 1:2.500 in 0.5%BSA-PBS-Tween was incubated for 1h at 37°C and then for 1h at 4°C. Plates were washed 5 times with 200µl/well of PBS-Tween followed by adding 100µl/well ABTS buffer and then were read after 5min, 10min, 15 min, 20min at OD 405 and 495 on ELISA reader CLARIOstar (BMG Labtech, Germany). Each patient`s serum was diluted 1:2 and 1:4 (if ImmunoCAP result for this sample were less than 50 kU_A/L) or 1:8 and 1:16 (if ImmunoCAP result for this sample were more than 50 kU_A/L). Using the formula $y=-60.302-50.933x-30.103x^3 +60,7969e^x$ for each OD sample obtained by ELISA we calculated the IgE concentration in ng/ml. Values with OD>2.8 after 10 min were excluded from the further calculations since curve was already reaching the plateau. Considering the two dilutions for each serum sample and exclusion of samples with OD>2.8, we obtained 34 samples for IgE reactivity testing to Bet v 1. For comparison, IgE levels to Bet v 1 for the same patients` sera were determined using quantitative IgE ImmunoCAP (Phadia 100) and by ImmunoCAP ISAC chip (Thermofisher, Uppsala, Sweden) according to manufacturer`s instructions.

A dilution series of IgEmoAb (2.42 µg/ml, 242 ng/ml, 24.2 ng/ml, 2.42 ng/ml) were also tested using ImmunoCAP technology. We noted that the ImmunoCAP determination of the concentrations of IgEmoAb were different (i.e., yielding approx. 30%) from the results obtained by microBCA protein determination which may be due to differences between the ImmunoCAP IgE standard and IgEmoAB. According to the factor $1kU_A/l=7$ ng /ml (i.e., real conversion

factor) obtained by measuring the IgEmoAb dilution by ImmunoCAP, the calculation for conversion of ImmunoCAP results from kU_A/L to ng/ml were made.

Using a set of sera (n=19) from clinically well-characterized cat allergic patients (supplementary Table 3) Fel d 1-specific IgE levels were quantified with the IgE ELISA as described above and compared with results obtained by quantitative IgE ImmunoCAP measurements. Considering the two dilutions for each serum sample and exclusion of samples with OD>2.8, we obtained 37 samples for IgE reactivity testing to Fel d 1. An OD of 0.05 was determined as a cut-off value for specific IgE reactivity by ELISA using serum dilutions from a non-allergic blood donor.

Measurement of allergen-specific IgE levels by ImmunoCAP and ImmunoCAP ISAC technology

In order to compare IgE levels determined by ImmunoCAP and the quantitative IgE ELISA, exactly the same recombinant allergen preparations (i.e., rBet v 1, rFel d 1) were used for the ELISA and for preparing ImmunoCAPs using Streptavidin CAPs as described [S5]. Two µg of biotin-labelled recombinant allergen rBet v 1 or rFel d 1 were added to the Streptavidin CAPs and incubated 30 minutes at RT. 150 µl of sera from 19 patients with cat allergy and 19 patients with birch pollen allergy and also set of dilutions of IgEmoAb were tested by ImmunoCAP Phadia-100 machine as described by the manufacturer (Thermo Fisher Scientific, Waltham, USA). Nineteen patients with birch pollen allergy were additionally tested by ImmunoCAP ISAC technology according to manufacturer`s instructions (Thermo Fisher Scientific, Waltham, USA).

Statistical analysis

Correlation between ELISA results, ImmunoCAP and ImmunoCAP ISAC chip were assessed using Spearman's correlation coefficient. Correlation coefficients (r), regression equations, and statistical significances (p values) were determined (SPSS 11.5 for Windows, Spearman Correlation). A p value <0.05 was considered significant.

References:

S1. Laffer, S. *et al.* Molecular characterization of Bip 1, a monoclonal antibody that modulates IgE binding to birch pollen allergen, Bet v 1. *J. Immunol.* **157**, 4953–62 (1996).

S2. Laffer, S. *et al.* A molecular model of type I allergy: identification and characterization of a nonanaphylactic anti-human IgE antibody fragment that blocks the IgE-FcepsilonRI interaction and reacts with receptor-bound IgE. *J. Allergy Clin. Immunol.* **108**, 409–16 (2001).

S3. Lupinek, C. *et al.* Trimolecular complex formation of IgE, FcεRI, and a recombinant nonanaphylactic single-chain antibody fragment with high affinity for IgE. *J. Immunol.* **182**, 4817–4829 (2009).

S4. Grönlund, H. *et al.* Formation of disulfide bonds and homodimers of the major cat allergen Fel d 1 equivalent to the natural allergen by expression in Escherichia coli. *J. Biol. Chem.* **278**, 40144–51 (2003).

S5. Erwin, E. A. *et al.* Quantitative measurement of IgE antibodies to purified allergens using streptavidin linked to a high-capacity solid phase. *J. Allergy Clin. Immunol.* **115**, 1029–35 (2005).

Supplementary Figures and Tables

Supplementary Figure 1. Coomassie-stained SDS-PAGE of purified IgEmoAb under non-reducing and reducing conditions. M, molecular weight marker (kDa) (**A**). Nitrocellulose-blotted IgEmoAb under non-reducing and reducing conditions was detected by anti-human IgE-HRP. Molecular weights are indicated on the left margins (**B**) Analysis of IgEmoAb by circular dichroism. The mean residue molar ellipticities (*y*-axis: degree cm^2 $dmol^{-1}$) were recorded for a range of wavelengths (*x*-axis) (**C**).

Supplementary Figure S2. Recording of standard curves at different time points. A series of dilutions of IgEmoAb (x-axis: ng/ml) was reacted with ELISA plate-bound rBet v 1 and IgE binding (y-axis: optical density (OD) values corresponding to bound IgE) was measured at different time points (5, 10, 15 and 20 min).

Supplementary Table S1. Demographic and clinical characterization of birch pollen (B1-B19) and cat allergic (C1-C19) patients.

Supplementary Table S2. Bet v 1-specific IgE levels determined by ELISA, ImmunoCAP and ImmunoCAP ISAC for birch pollen allergic patients

Supplementary Table S3. Fel d 1-specific IgE levels determined by ELISA, ImmunoCAP and ImmunoCAP ISAC for cat allergic patients

Supplementary Table S4. Fel d 1-specific IgE levels determined by ELISA shown as optical density (OD) values for cat allergic patient's sera in dilutions 1:2 and 1:4 or 1:8 and 1:16 for C3.

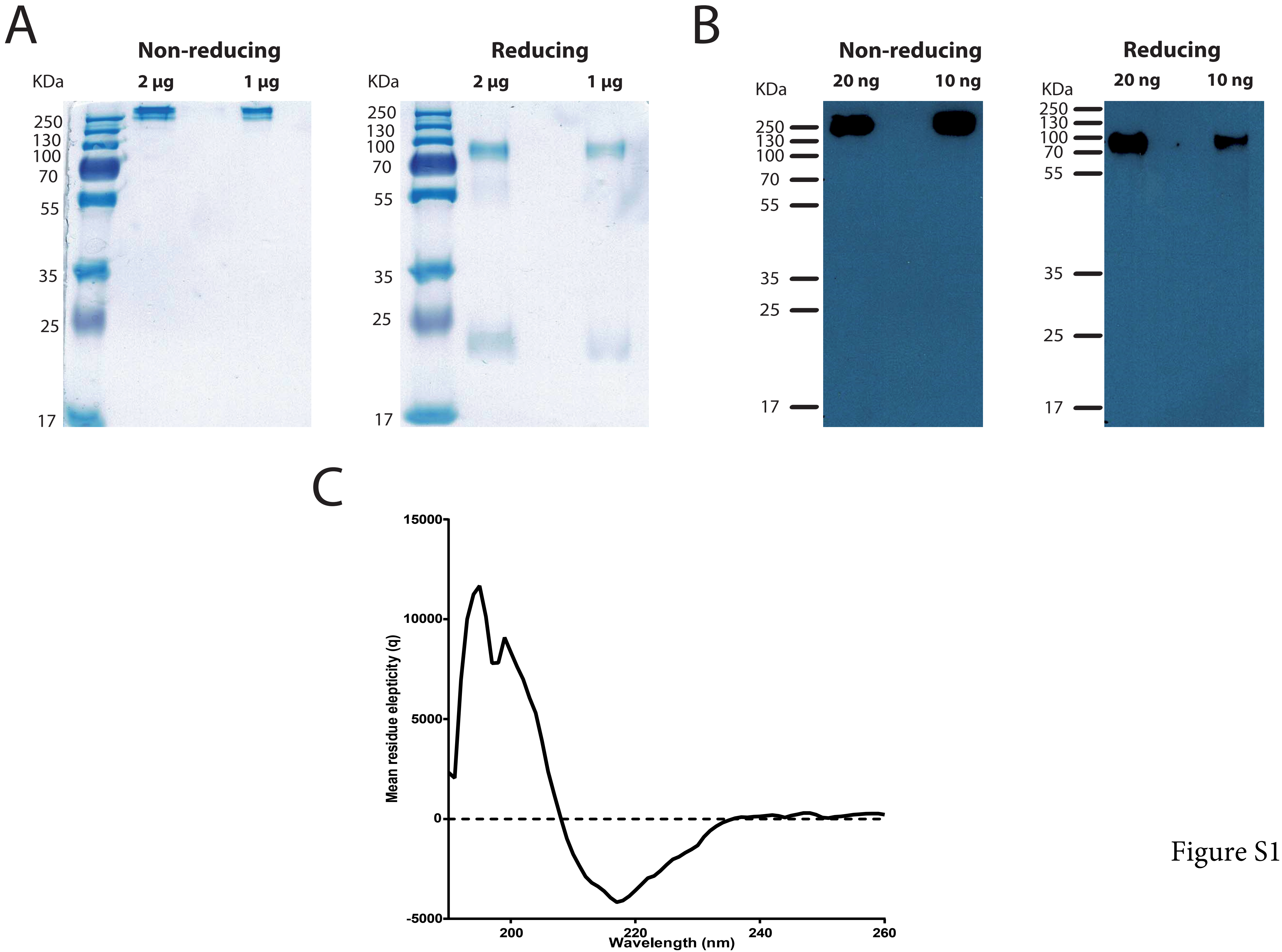
A
Non-reducing
Reducing
KDa
2 µg
1 µg
250
130
100
70
55
35
25
17
B
Non-reducing
Reducing
20 ng
10 ng
C
15000
10000
5000
0
-5000
Mean residue elepticity (q)
200
220
240
260
Wavelength (nm)

Figure S1

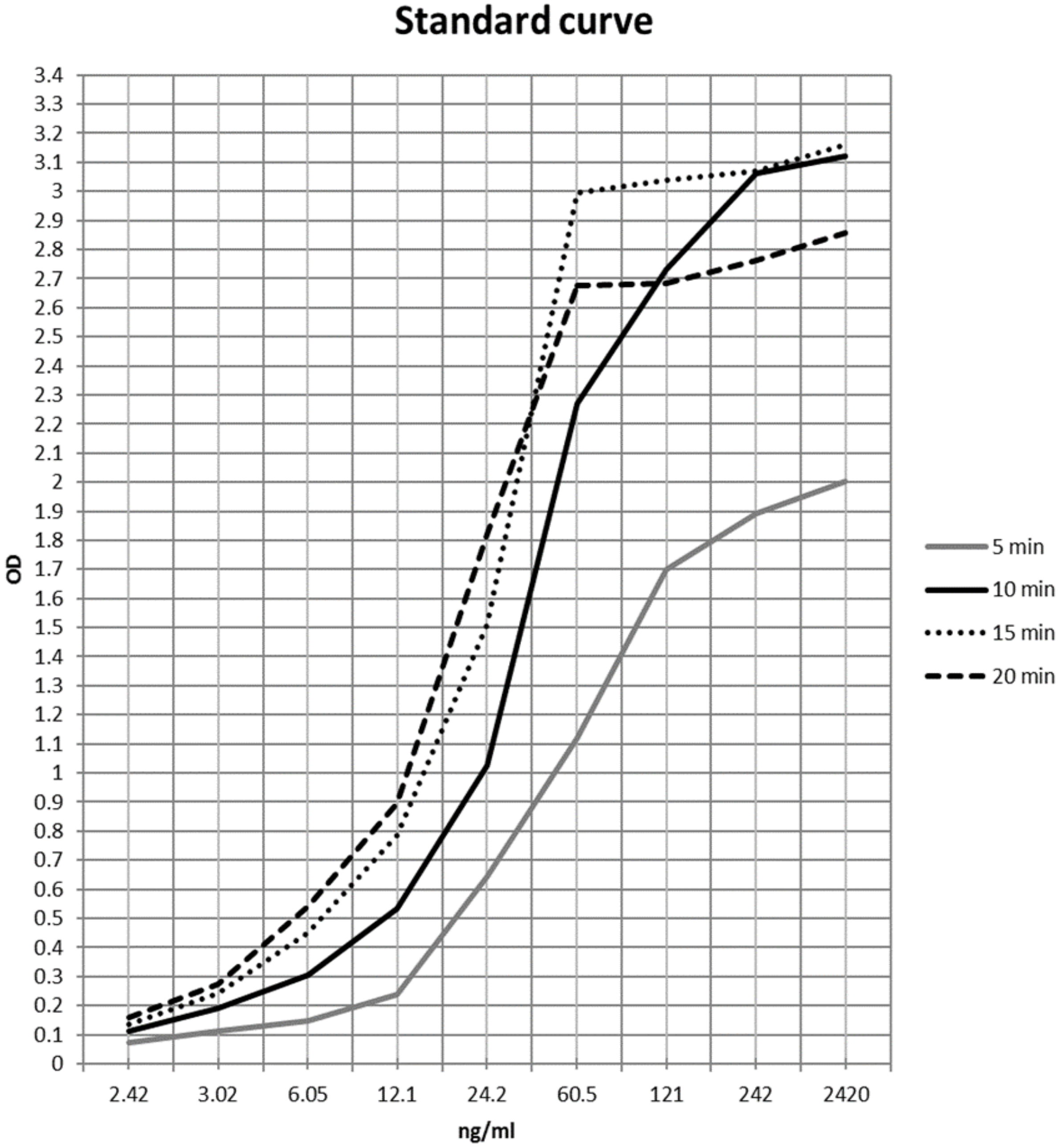

Supplementary Figure S2. Recording of standard curves at different time points. A series of dilutions of IgEmoAb (x-axis: ng/ml) was reacted with ELISA plate-bound rBet v 1 and IgE binding (y-axis: optical density (OD) values corresponding to bound IgE) was measured at different time points (5, 10, 15 and 20 min).

Supplementary Table S1. Demographic and clinical characterization of birch pollen (B1-B19) and cat allergic (C1-C19) patients.

	Sex	Age	Allergy clinical symptoms				Other allergies				
			Asthma	Rhinitis	Conj.	Dermat.	Dust	Pet	Pollen	Food	Mold
**Patients with Birch Pollen Allergy**											
B1	m	10	neg	pos	pos	neg	neg	pos	pos	pos	pos
B2	m	10	pos	pos	pos	pos	neg	pos	pos	neg	neg
B3	m	14	neg	pos	pos	neg	pos	pos	pos	pos	pos
B4	m	15	pos	pos	pos	pos	pos	pos	pos	neg	pos
B5	f	16	neg	pos	pos	neg	neg	pos	pos	pos	neg
B6	m	12	neg	pos	pos	neg	neg	pos	pos	pos	neg
B7	f	15	neg	pos	pos	neg	pos	pos	pos	pos	neg
B8	f	12	neg	pos	pos	pos	neg	pos	pos	pos	neg
B9	m	10	neg	pos	pos	neg	neg	pos	pos	neg	neg
B10	m	11	pos	pos	pos	neg	neg	pos	pos	pos	pos
B11	m	15	neg	pos	pos	neg	pos	pos	pos	neg	neg
B12	f	11	pos	pos	pos	pos	neg	pos	pos	pos	neg
B13	m	15	pos	pos	pos	neg	pos	pos	pos	neg	neg
B14	f	10	pos	pos	pos	pos	pos	pos	pos	pos	neg
B15	f	11	neg	pos	pos	neg	neg	pos	pos	neg	neg
B16	f	11	neg	pos	pos	neg	neg	pos	pos	neg	neg
B17	f	11	pos	pos	pos	neg	neg	pos	pos	neg	neg
B18	m	10	neg	pos	pos	pos	neg	pos	pos	neg	neg
B19	f	15	neg	pos	pos	pos	pos	pos	pos	pos	neg
**Patients with Cat Allergy**											
C1	m	29	pos	neg	pos	neg	pos	pos	pos	pos	pos
C2	m	26	pos	pos	pos	pos	pos	pos	pos	n/d	n/d
C3	f	25	pos	pos	pos	pos	pos	pos	pos	n/d	pos
C4	f	62	pos	pos	pos	pos	n/d	n/d	n/d	n/d	n/d
C5	f	35	pos	pos	pos	pos	pos	pos	pos	n/d	n/d
C6	f	27	pos	pos	pos	pos	pos	pos	pos	n/d	n/d
C7	m	21	pos	pos	pos	neg	pos	pos	pos	n/d	n/d
C8	m	29	neg	pos	pos	pos	pos	pos	neg	n/d	n/d
C9	m	24	pos	pos	pos	pos	pos	pos	pos	pos	n/d
C10	f	57	neg	pos	pos	neg	pos	pos	pos	n/d	n/d
C11	m	47	neg	pos	pos	neg	neg	neg	pos	n/d	n/d
C12	m	20	pos	pos	pos	neg	pos	pos	pos	n/d	n/d
C13	m	31	pos	pos	neg	neg	neg	pos	n/d	n/d	neg
C14	m	20	pos	pos	pos	neg	pos	pos	pos	pos	n/d
C15	m	22	pos	pos	neg	neg	pos	pos	neg	n/d	n/d
C16	m	20	pos	pos	neg	neg	pos	pos	pos	pos	n/d
C17	f	38	neg	pos	pos	neg	pos	pos	pos	n/d	n/d
C18	f	40	pos	pos	pos	neg	n/d	n/d	n/d	n/d	n/d
C19	m	18	pos	pos	pos	neg	pos	pos	pos	n/d	n/d

Supplementary Table S2. Bet v 1-specific IgE levels determined by ELISA, ImmunoCAP and ImmunoCAP ISAC for birch pollen allergic patients (B1-B19)

Patient ID	OD Mean	ng/ml	ImmunoCAP kU_A/L	ISAC ISU
B1	2.209	902.4	346	139.9
B2	0.867	329.6	64	11.1
B3	0.828	313.6	62.3	52.5
B4	0.594	212.8	60.5	57.2
B5	0.859	326.4	59.7	59.9
B6	1.014	97.2	48	45.8
B7	0.88	334.4	75.7	78.2
B8	2.595	1536	331	153.5
B9	1.035	396.8	16.1	20.3
B10	1.576	142.4	46	22.7
B11	0.59	52.4	9.69	16.3
B12	1.058	101.2	42.1	32.8
B13	0.268	20	3.07	3.7
B14	0.943	90	33	11.4
B15	2.742	1966.4	430	148.1
B16	0.306	23.2	2.64	4.1
B17	0.199	14	1.68	3.3
B18	1.238	467.2	146	52.7
B19	2.703	1838.4	342	144.3

Supplementary Table S3. Fel d 1-specific IgE levels determined by ELISA and ImmunoCAP for cat allergic patients (C1-C19) and non-allergic donor (D1)

Patient ID	OD Mean	ng/ml	ImmunoCAP kU_A/L
C1	0.703	64.8	5.20
C2	1.816	162.8	34.50
C3	1.041	398.4	57.20
C4	0.059	4.8	0.03
C5	0.059	4.8	0.08
C6	0.687	63.2	8.79
C7	0.621	56	5.43
C8	0.153	10.8	1.76
C9	1.143	108.8	16.30
C10	0.418	34.4	2.55
C11	0.064	4.8	0.10
C12	0.061	4.8	0.08
C13	0.534	46.4	3.19
C14	0.115	8	1.60
C15	0.069	5.2	0.16
C16	0.145	10	1.34
C17	0.573	50.8	4.50
C18	2.331	260.8	22.80
C19	0.703	368	29.00

Donor ID			
D1	0.043		0.001

Supplementary Table S4. Fel d 1-specific IgE levels determined by ELISA shown as optical density (OD) values for cat allergic patient's sera in dilutions 1:2 and 1:4 or 1:8 and 1:16 for C3.

Patient ID	Serum Dilution	OD Mean	ng/ml
C1	1:2	1.262	59.4
C1	1:4	0.703	64.8
C2	1:2	2.734	242.6
C2	1:4	1.816	162.8
C3	1:8	2.100	403.2
C3	1:16	1.041	398.4
C4	1:2	0.067	2.6
C4	1:4	0.059	4.8
C5	1:2	0.075	2.8
C5	1:4	0.059	4.8
C6	1:2	1.090	52
C6	1:4	0.687	63.2
C7	1:2	1.131	54
C7	1:4	0.621	56
C8	1:2	0.264	9.8
C8	1:4	0.153	10.8
C9	1:2	2.057	97
C9	1:4	1.143	108.8
C10	1:2	0.491	21
C10	1:4	0.418	34.4
C11	1:2	0.091	3.2
C11	1:4	0.064	4.8
C12	1:2	0.062	2.4
C12	1:4	0.061	4.8
C13	1:2	0.779	36.6
C13	1:4	0.534	46.4
C14	1:2	0.156	5.4
C14	1:4	0.115	8.00
C15	1:2	0.082	3.00
C15	1:4	0.069	5.20
C16	1:2	0.189	6.6
C16	1:4	0.145	10.00
C17	1:2	0.879	41.8
C17	1:4	0.573	50.8
C18	1:2	2.695	226.6
C18	1:4	2.3331	260.8
C19	1:2	2.979	375.8
C19	1:4	2.569	368
Donors ID			
D1	1:2	0.045	
D1	1:4	0.043	
