# Supplementary figures and images for: Highly sensitive ELISA‐based assay for quantification of allergen‐specific IgE antibody levels
Source: Allergy. 2020 May 27;75(10):2668–70. doi: 10.1111/all.14325 (PMC7687237; doi:10.1111/all.14325)

## Slide 1
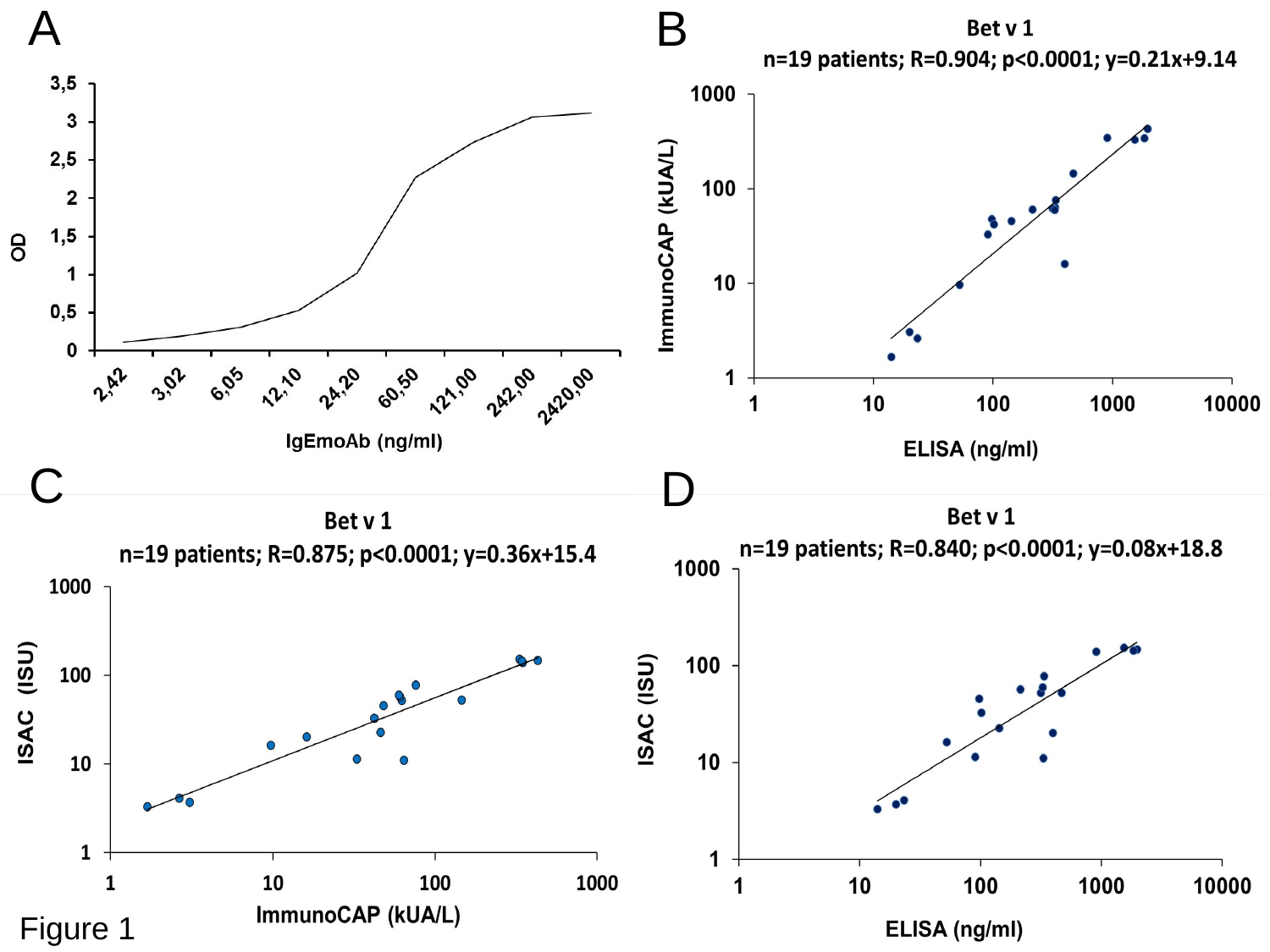

A
B
C
D
Figure 1

Supplement: Supplementary file 2 [file ALL-75-2668-s002.pptx]

## Slide 1
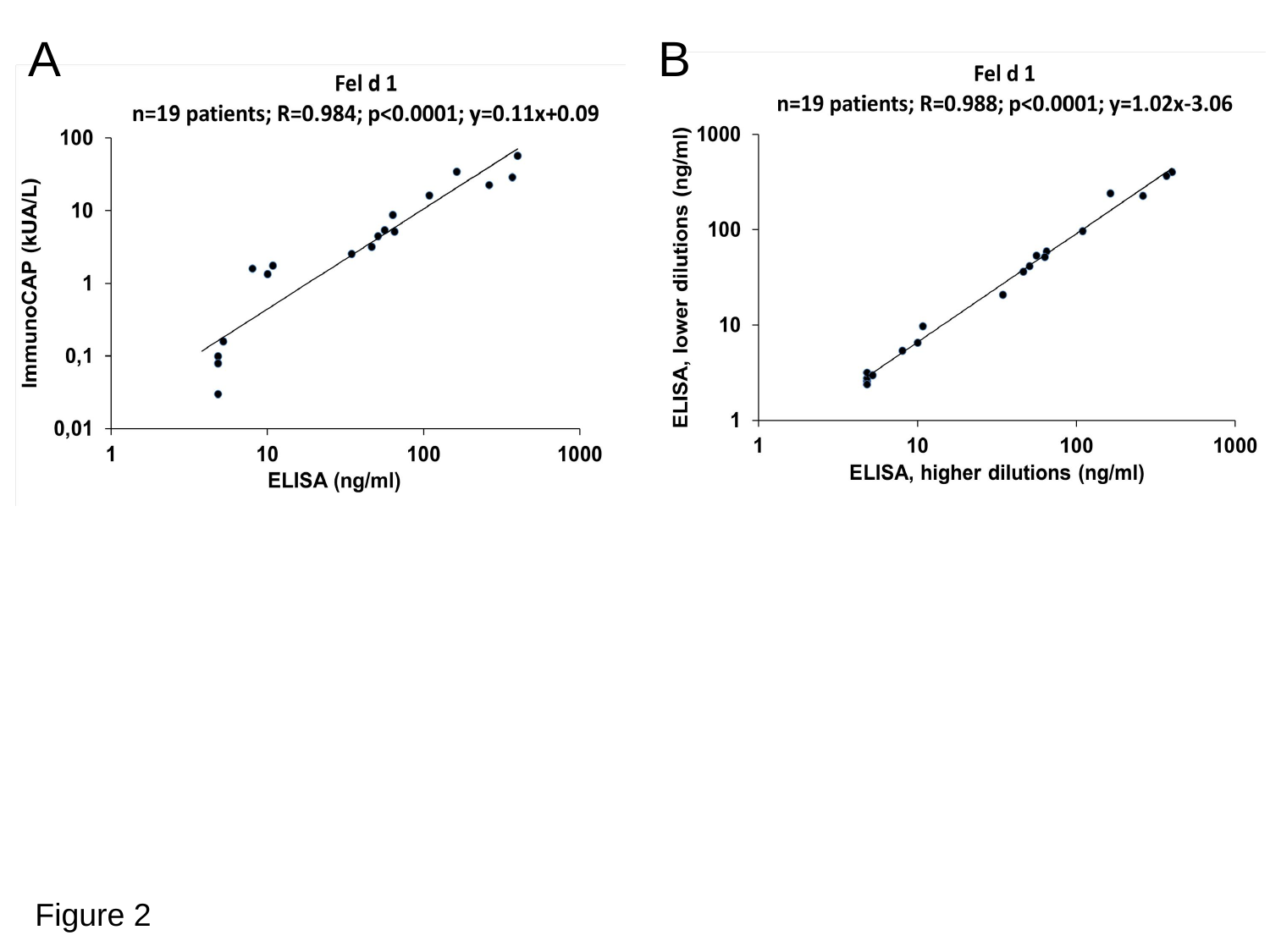

A
B
Figure 2

Supplement: Supplementary file 3 [file ALL-75-2668-s003.pptx]
